# Branch site mutated mice revealed distinct roles of two Runx2 isoforms in bone development

**DOI:** 10.3389/fcell.2026.1770176

**Published:** 2026-04-24

**Authors:** Qing Jiang, Manyu Zhang, Haoyunyan Jin, Ziheng Zhang, Chiharu Sakane, Yuki Matsuo, Simeng Lai, Wen Zhang, Huilin Yang, Toshihisa Komori, Xin Qin

**Affiliations:** 1 Institute of Orthopaedics, Suzhou Medical College, Soochow University, Suzhou, China; 2 Research Center for Biomedical Models and Animal Welfare, Nagasaki University Graduate School of Biomedical Sciences, Nagasaki, Japan; 3 Department of Molecular Tumor Biology, Nagasaki University Graduate School of Biomedical Sciences, Nagasaki, Japan; 4 Department of Skeletal Development and Regenerative Biology, Nagasaki University Graduate School of Biomedical Sciences, Nagasaki, Japan; 5 Department of Orthopaedics, The First Affiliated Hospital of Soochow University, Suzhou, China

**Keywords:** cleidocranial dysplasia, endochondral ossification, osteoblast differentiation, *Runx2-*I, *Runx2-*II, suture mesenchymal cell proliferation

## Abstract

Runx2 is a key regulator of osteoblast differentiation and chondrocyte maturation. However, the distinct functions of the two functional isoforms remain to be clarified. Transcription of two isoforms, Runx2-I and Runx2-II, starts from the proximal and distal promoters upstream of exons 2 and 1, respectively. To investigate the functions of the two isoforms, we generated a novel mouse model (*Runx2*-br^mut/mut^), in which intron 1 splicing for *Runx2*-II was disrupted by mutating the branch site essential for splicing. *Runx2*-II was severely reduced but *Runx2*-I was increased in *Runx2*-br^mut/mut^ mice as compared with those in *Runx2*-br^wt/wt^ mice. Although *Runx2*-II was about three times higher than *Runx2*-I in E15.5 limbs and newborn calvaria of *Runx2*-br^wt/wt^ mice, *Runx2*-II was extremely lower than *Runx2*-I in *Runx2*-br^mut/mut^ mice. Endochondral ossification was retarded in *Runx2*-br^mut/mut^ mice, but the delay was milder than in *Runx2* ^+/−^ mice, and the primary spongiosa formation was impaired due to the reduced osteoblasts. The development of calvaria in the newborn was retarded similar to *Runx2* ^+/−^ mice, which showed cleidocranial dysplasia, but it was much less affected than in *Runx2* ^+/−^ mice at 8 weeks of age, and the suture mesenchymal cell proliferation increased. Furthermore, clavicle development was less affected than that in *Runx2* ^+/−^ mice throughout their lives. The trabecular and cortical bones in the femurs of *Runx2*-br^mut/mut^ mice were lower than those of *Runx2*-br^wt/wt^ mice owing to the reduced bone formation, and the strength of the bones was also weaker. Osteoblast differentiation was impaired in *Runx2*-br^mut/mut^ mice. Overexpression of *Runx2*-II failed to affect endogenous *Runx2* expression *in vitro*, and *Runx2* knockdown by siRNA failed to affect the proximal promoter activity. These findings indicated that both isoforms contribute to endochondral ossification, *Runx2*-I can compensate for *Runx2*-II in endochondral ossification, Runx2-II plays important roles in osteoblast differentiation, and Runx2-I plays important roles in the development of calvaria and clavicles, at least in part, by enhancing suture mesenchymal cell proliferation. Our findings also showed that a minimal amount of Runx2-II is necessary for the efficient function of Runx2-I, indicating a basal requirement of Runx2-II in bone development, but that *Runx2* expression is not autoregulated by Runx2.

## Graphical Abstract

**Figure:**
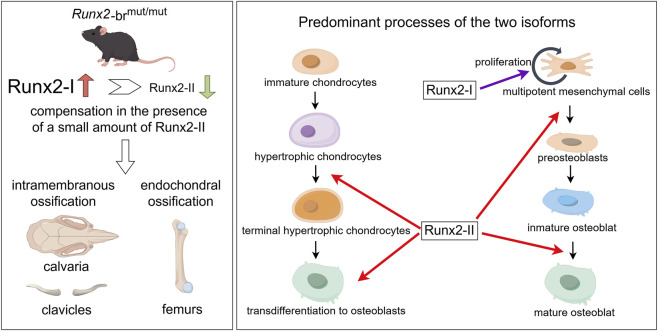

## 1 Introduction

Runt-related transcription factor 2 (Runx2) is an essential transcription factor for skeletal development. Runx2 drives osteoblast precursor cell proliferation, osteoblast differentiation, chondrocyte maturation, and the transdifferentiation of hypertrophic chondrocytes into osteoblasts (Komori, 2022; Kawane et al., 2018; Komori et al., 1997; Otto et al., 1997; Yoshida et al., 2004; Qin et al., 2020; Qin et al., 2021). Germline deletion of *Runx2* in mice results in lethality at birth due to the complete absence of bone formation (Komori et al., 1997; Otto et al., 1997). In humans, heterozygous mutations in *RUNX2* cause cleidocranial dysplasia (CCD), characterized by hypoplastic clavicles, open fontanelles, supernumerary teeth, and short stature. Similarly, *Runx2* ^+/−^ mice exhibit hypoplastic clavicles, open fontanelles, and mild osteopenia, which are key features of CCD (Otto et al., 1997; Mundlos et al., 1997; Qin et al., 2019; Jiang et al., 2022). Furthermore, gain-of-function variants and overexpression of *RUNX2* have been identified in patients with craniosynostosis (Cuellar et al., 2020).

**FIGURE 1:**
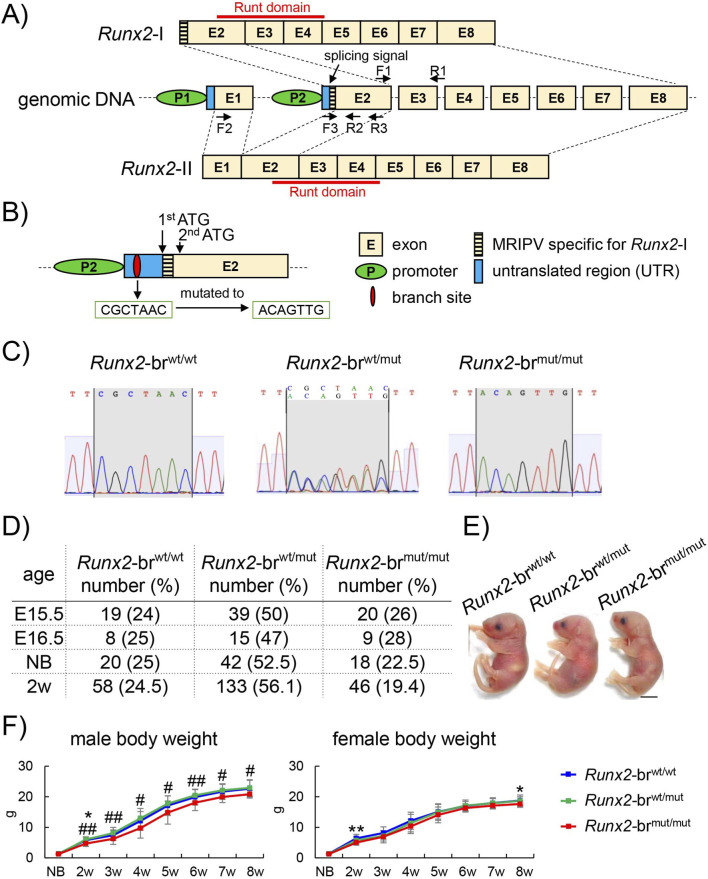
Strategies for generating *Runx2*-br^mut/mut^ mice and genotyping, survival rates, appearance of newborns, and body weights of *Runx2*-br^wt/wt^, *Runx2*-br^wt/mut^, and *Runx2*-br^mut/mut^ mice. **(A)** Schematic presentation of *Runx2*-I and *Runx2*-II mRNAs in *Runx2*-br^wt/wt^ mice and primer designs for amplifying total *Runx2* (F1–R1), *Runx2*-I specific transcripts (F3–R2 and F3–R3), and *Runx2*-II specific transcripts (F2–R2). **(B)** Schematic of the construction strategy for *Runx2*-br^mut/mut^ mice. **(C)** Genotyping by genome sequencing of *Runx2*-br^wt/wt^, *Runx2*-br^wt/mut^, and *Runx2*-br^mut/mut^ mice. **(D)** Survival rates of *Runx2*-br^wt/wt^, *Runx2*-br^wt/mut^, and *Runx2*-br^mut/mut^ mice. **(E)** Appearance of *Runx2-*br^wt/wt^, *Runx2*-br^wt/mut^, and *Runx2*-br^mut/mut^ newborns. Scale bars: 0.5 cm. **(F)** Body weight curves from newborns (NB) to 8 weeks of age. The number of mice analyzed: newborn, n = 14 (*Runx2*-br^wt/wt^), n = 33 (*Runx2*-br^wt/mut^), and n = 12 (*Runx2*-br^mut/mut^); 2–8 weeks of age, males: n = 16 (*Runx2*-br^wt/wt^), n = 33 (*Runx2*-br^wt/mut^), and n = 12 (*Runx2*-br^mut/mut^); females: n = 21 (*Runx2*-br^wt/wt^), n = 40 (*Runx2*-br^wt/mut^), and n = 11 (*Runx2*-br^mut/mut^). *Versus *Runx2*-br^wt/wt^ mice, ^#^versus *Runx2*-br^wt/mut^ mice, *^,#^p < 0.05, **^,##^p < 0.01.

*Runx2* is transcribed from two distinct promoters, the distal (P1) and proximal (P2) promoters, generating isoforms with unique N-termini. The type II Runx2 isoform (Runx2-II), driven by P1, contains the N-terminal specific 19 amino acids (MASNS) encoded in exon 1, whereas the type I Runx2 isoform (Runx2-I), driven by P2, contains the N-terminal specific 5 amino acids (MRIPV) encoded by exon 2. Both isoforms share 509 amino acids encoded by exons 2–8, which include the critical DNA-binding domain, runt (Figure 1A) (Komori, 2018).

To investigate the isoform-specific functional contributions to intramembranous and endochondral ossification, two *Runx2*-II deficient mouse models were established. Both the P1 promoter and exon 1 were replaced with a neo gene in the first model (*Runx2*-II^−/−^ mice), whereas only exon 1 was replaced with Lacz and neo, leaving the P1 promoter in the second model (*Runx2*-II^lacz/lacz^ mice) (Xiao et al., 2004; Ueta et al., 2001). The former model displayed severe defects in both endochondral and intramembranous ossification, and 20% of the mice survived after birth, showing profound osteopenia (Xiao et al., 2004; Xiao et al., 2005), whereas the latter model exhibited milder osteopenia with a normal lifespan (Liu et al., 2011). Both model mice completely lacked *Runx2*-II, but *Runx2*-I was upregulated in *Runx2*-II^−/−^ mice but not in *Runx2*-II^lacz/lacz^ mice. As the osteopenia in *Runx2*-II^−/−^ mice was much severer than that in *Runx2*-II^lacz/lacz^ mice, the phenotypes of *Runx2*-II deficient mice were controversial. Further, even in *Runx2*-II^−/−^ mice, their phenotypes were much milder than those of *Runx2* ^−/−^ mice, indicating that Runx2-I plays an important role in bone development. However, the attempt to generate *Runx2*-I^−/−^ mice was unsuccessful. Homozygous mutation of the first ATG to a stop codon in exon 2 resulted in no apparent phenotypes, suggesting that the protein translated from the second ATG in exon 2 has a similar physiological activity to that from the first ATG (Runx2-I) (Komori, 2018; Okura et al., 2014). Currently, no appropriate *Runx2*-I^−/−^ mouse model is available for comprehensive functional analysis. Therefore, the functional differences of Runx2-I and Runx2-II remain to be clarified.

In two *Runx2*-II deficient mouse models, the absence of both P1 and exon 1 (*Runx2*-II^−/−^ mice) and presence of P1 but absence of exon 1 (*Runx2*-II^lacz/lacz^ mice) showed different phenotypes. Therefore, we tried to interrupt the generation of *Runx2*-II keeping the P1 and exon 1 genomic region intact to investigate the functional differences of the two isoforms. The branch site, located 18–40 nucleotides upstream of the 3’ (acceptor) splice site, serves as a critical recognition element for spliceosome assembly. By mutating the branch site in intron 1, we attempted to disrupt the splicing between exons 1 and 2 and interrupt the *Runx2*-II formation. In the branch site mutated (*Runx2*-br^mut/mut^) mice, splicing of the first intron was impaired, leading to a drastically reduced expression of *Runx2*-II and an increased *Runx2*-I expression in cartilage and bone tissues. Here, we show the functional properties of Runx2-I and Runx2-II, which were revealed in *Runx2*-br^mut/mut^ mice.

## 2 Materials and methods

### 2.1 Generation of *Runx2*-br^mut/mut^ mice

The branch site in *Runx2* intron 1, located 31-bp upstream of the acceptor splicing site of exon 2, was mutated from CGCTAAC to ACAGTTG. A DNA fragment containing the mutated branch site flanked by 131-bp 5′ homology and 67-bp 3′ homology was synthesized. The DNA fragment, two guide RNAs targeting the sequences CCG​GCC​ACT​TCG​CTA​ACT​TGT​GG and ACA​GCC​ACA​AGT​TAG​CGA​AGT​GG, and Cas9 mRNA were injected into fertilized C57BL/6J mouse eggs. The mice were generated by Shanghai Model Organisms Center, Inc. Because only 7-bp nucleotides were mutated, a specific sequencing-based genotyping strategy was used. Genomic DNA was extracted from the mouse toe or tail using the Steady Pure Universal Genomic DNA Extraction Kit (Hunan Accurate Bio-Medical Technology Co., Ltd., Changsha, China). A 659-bp fragment containing the mutation site was amplified by PCR using the forward primer GCC​CCT​ACT​GCA​AGC​TGT​TA and reverse primer TCC​GCG​ATG​ATC​TCC​ACC​AT, and the forward primer was used for sequencing of the PCR product. Sequencing was performed by Sangon Biotech Co., Ltd. (Shanghai, China). *Runx2*-br^wt/mut^ mice were crossed with C57BL/6J mice, and *Runx2*-br^mut/mut^ mice were generated by brother-sister mating. Mice were maintained on a C57BL/6J background. Runx2 heterozygous (*Runx2* ^+/−^) mice were generated as previously described (Komori et al., 1997). *Runx2* ^+/−^ mice were backcrossed with C57BL/6N mice more than 12 times. Prior to the initiation of the study, all animal experiments were approved by the Ethics Committee of Soochow University (202307A0519) and conducted in accordance with the National Institutes of Health (NIH) guidelines. The mice were housed in groups of no more than five per cage in a pathogen-free environment on a 12-h light cycle at 22 °C ± 2 °C with free access to water.

### 2.2 Real-time reverse-transcription polymerase chain reaction (RT-PCR) and western blot analyses

Total RNA was extracted from the hindlimbs or calvaria of *Runx2*-br^wt/wt^, *Runx2*-br^wt/mut^, and *Runx2*-br^mut/mut^ mice at E15.5 and at the newborn stage. In 8-week-old mice, the muscle tissue was removed, tibiae were cut off at the metaphyses, the bone marrow was flushed out with PBS, and the remaining bone tissue was used for RNA extraction. The tissues were homogenized using TRIzol (Life Technologies Corporation, Carlsbad, California, USA). Total RNA was extracted from the supernatant of the centrifuged mixture of TRIzol and chloroform, and proteins were obtained from the lower layer. Real-time RT-PCR was performed using the HiScript® III RT SuperMix for qPCR and Taq Pro Universal SYBR qPCR Master Mix (Vazyme Biotech, Nanjing, China). Primer sequences are listed in Supplementary Table S1. The primer set for *Bglap/Bglap2* detection was designed to detect both *Bglap* and *Bglap2*. We normalized the values obtained to those of *Actb* using the 2^(−delta delta C(t))^ method. Western blotting was performed using monoclonal rabbit anti-Runx2 (Cell Signaling, Danvers, MA, USA) and anti-β-actin (Cell Signaling).

### 2.3 Droplet digital PCR

The absolute quantities of *Runx2*-I and *Runx2*-II mRNA were measured using the Sniper DQ24 Digital PCR System (Sniper, Suzhou, China). The PCR reaction of cDNA was performed in a 20 μL volume containing 10 μL of 2×T5 Fast qPCR Mix (Probe), 0.7 μL of 10 μM primers, 0.6 μL of 10 μM probes, 2 μL of DNA template, 0.4 μL of 50×ROX Reference Dye I, and sterile water to reach the final volume. The thermal cycler settings were 1 cycle at 60 °C for 5 min and 95 °C for 2 min, followed by 40 cycles at 95 °C for 15 s and 60 °C for 1 min. After PCR amplification, fluorescence was detected in each droplet to identify positive and negative signals. The number of positive and negative droplets was counted, and the absolute nucleic acid concentration in the original sample was calculated based on the Poisson distribution and known droplet volume. Droplet digital PCR technical services were provided by Tsingke Biotech (Beijing, China).

### 2.4 Skeletal preparation and histological analysis

Skeletal preparation of embryos at E15.5 and newborns was performed as previously described (Komori et al., 1997). Hindlimbs from embryonic and neonatal mice were dissected and fixed oscillatory in 4% PFA at 4 °C overnight. The mice were euthanized and perfusion-fixed with 4% PFA at 7 days and 3 weeks of age, and the femurs were isolated and fixed. After decalcification in 10% EDTA, the bone samples were embedded in paraffin. Four-micrometer sections were stained with hematoxylin and eosin (H&E) (Biosharp, Anhui, China) or Safranin O (Solarbio, Beijing, China). Immunohistochemistry was performed using a monoclonal rabbit anti-Runx2 antibody (Cell Signaling), polyclonal rabbit anti-Col1a1 antibody (Merck Millipore, Billerica, MA, USA), and a rabbit two-step test kit (ZSGB-BIO, Beijing, China). No significant signals were observed in the negative control experiments performed without the primary antibody. Tartrate-resistant acid phosphatase (TRAP) staining was performed using Fast Red Violet LB salt (Absin, Shanghai, China). Immunostained sections were counterstained with methyl green (Beyotime Biotechnology, Shanghai, China), and TRAP-stained sections were counterstained with hematoxylin. To analyze EdU incorporation, 7-day-old mice were injected subcutaneously with EdU at a dose of 100 μg/g body weight 2 h before sacrifice. EdU incorporation was detected using an EdU staining kit (Beyotime Biotechnology, Shanghai, China), and the sections were counterstained with hematoxylin (Beyotime Biotechnology).

### 2.5 Micro-computed tomography (micro-CT) analysis

The left femurs, devoid of muscle tissue, were stored in 70% ethanol solution. Micro-CT analysis was performed using a Skyscan1176 (Bruker, Aartselaar, Belgium). Scanned slice data were used for three-dimensional analysis to calculate femoral morphometric parameters. Trabecular bone parameters were measured at the distal femoral metaphysis, scanning approximately 2.3 mm (0.5 mm from the growth plate) cranio-caudally, with 200 slices taken at 9-μm increments. For the femoral cortical bone, 20 slices were obtained at 9-μm increments. Trabecular and cortical bone parameters were analyzed using CTAn software (Bruker, Aartselaar, Belgium).

### 2.6 Dynamic bone histomorphometric analyses

For the assessment of dynamic histomorphometric indices, mice were subcutaneously injected with calcein at a dose of 0.2 mg/10 g body weight (dissolved in 1% NaHCO3) 5 and 2 days before euthanasia. The bone samples were subjected to gradient dehydration and embedded in a light-curing composite resin (Technovit 7200VLC; Kulzer-Exakt, Wehrheim, Germany). Subsequently, the bones were trimmed to the appropriate dimensions and ground down to a thickness of approximately 20-μm using a grinding stone and sandpaper. Bone formation parameters were measured using the ImageJ software.

### 2.7 Biomechanical test

The right humerus, devoid of muscle tissue, underwent a three-point bending test using a microcomputer-controlled electrical universal testing machine (HY–1080; Hengyi Instrument, Shanghai, China). A vertical load was applied to the midshaft at a constant rate of 3 mm/min with a support span of 6 mm until fracture occurred.

### 2.8 The differentiation of bone marrow-derived mesenchymal stem cells (BMSCs) and primary osteoblasts (POB) *in vitro*

BMSCs were isolated and cultured according to a published protocol (Huang et al., 2015), and POB were prepared as described previously (Qin et al., 2019). Cells were seeded in 48-well plates at 1.7 × 10^5^ cells/cm^2^ (BMSCs) or 1.1 × 10^5^ cells/cm^2^ (POB) in αMEM supplemented with 10% fetal bovine serum (Vazyme Biotech). The next day, the cells were confluent, and the medium was replaced with fresh medium containing the Inducer for Osteogenesis of Mouse Mesenchymal Stem Cells (Amizona Scientific, Birmingham, AL, USA). Staining for ALP (Beyotime Biotechnology) and mineralization (von Kossa) was performed at 3 and 9 days after confluence, respectively, as described previously (Jiang et al., 2024). RNA was collected from BMSCs and POB at 0, 3, and 9 days and 6 days after confluence, respectively.

### 2.9 Overexpression or knockdown of *Runx2* and reporter assay

For *Runx2* overexpression experiments, osteoblastic MC3T3-E1 cells were plated in 24-well plates at 1.6 × 10^4^cells/cm^2^ in αMEM supplemented with 10% fetal bovine serum. The next day, the cells were transfected with pME18S-FL3-enhanced green fluorescent protein (EGFP) or pME18S-FL3-*Runx2* using jetPRIME® (Polyplus, Paris, France), and RNA was extracted 48 h after transfection. The levels of total *Runx2*, including endogenous and exogenous *Runx2*, and those of endogenous *Runx2*-I, *Runx2*-II, and total *Runx2* were examined using real-time RT-PCR with the indicated primers (Figure 12A). For *Runx2* knockdown experiments, MC3T3-E1 cells were transfected with siRNA for the control or *Runx2* using the siRNA-mateplus transfection reagent (GenePharma, Suzhou, China), and RNA was extracted after 48 h. Two *Runx2* siRNAs were mixed and transfected into cells. The sense and antisense strands of the first *Runx2* siRNA were CCU​UGA​CCA​UAA​CAG​UCU​UTT and AAG​ACU​GUU​AUG​GUC​AAG​GTT, respectively, and those of the second *Runx2* siRNA were CCG​GGA​AUG​AUG​AGA​ACU​ATT and UAG​UUC​UCA​UCA​UUC​CCG​GTT, respectively. Total *Runx2* levels were examined using real-time RT-PCR with the indicated primers. *Runx2* 1.9-kb P2 (−1877-bp to −1-bp from the first ATG in exon 2) was PCR-amplified from the mouse genomic DNA. The 1.9-kb P2 fragment was cloned into the SacI-XhoI site of the pGL4.10- basic luciferase reporter vector (Promega, Madison, WI, USA) to generate the 1.9-kb P2-luc vector. Control siRNA-or *Runx2* siRNA-transfected MC3T3-E1 cells were further transiently transfected with pGL4.10 or 1.9-kb P2-luc and pRL-TK (Promega) using jetPRIME® (Polyplus, Paris, France). Transfected cells were cultured for 48 h, and luciferase activities were examined using the Dual-Luciferase Reporter Assay System (Vazyme Biotech) and normalized to Renilla luciferase activities.

### 2.10 Graphical abstract

The graphical abstract was drawn using Figdraw 2.0.

### 2.11 Statistical analysis

Values are expressed as the mean ± SD. Statistical analyses of two groups were performed using Student’s t-test, and those of more than three groups were conducted using ordinary one-way analysis of variance (ANOVA) using GraphPad Prism 8.0.1. Statistical significance was set at p < 0.05.

## 3 Results

### 3.1 Generation of *Runx2*-br^mut/mut^ mice

To disrupt the formation of *Runx2*-II, we mutated the branch site in intron 1 (CGCTAAC to ACAGTTG), which is located 31-bp upstream of the acceptor splicing site of exon 2 (Figures 1A,B), and the mutation was confirmed by sequencing (Figure 1C). Approximately 20% of *Runx2*-br^mut/mut^ mice died after birth (Figure 1D). The appearance of *Runx2*-br^wt/wt^, *Runx2*-br^wt/mut^, and *Runx2*-br^mut/mut^ mice was similar at the neonatal stage (Figure 1E). The body weights of male *Runx2*-br^mut/mut^ mice were lower than those of *Runx2*-br^wt/wt^ mice at 2 weeks of age and lower than those of *Runx2*-br^wt/mut^ mice at 2–8 weeks of age, while those of female *Runx2*-br^mut/mut^ mice were lower than those of *Runx2*-br^wt/wt^ mice at 2 and 8 weeks of age (Figure 1F).

**FIGURE 2:**
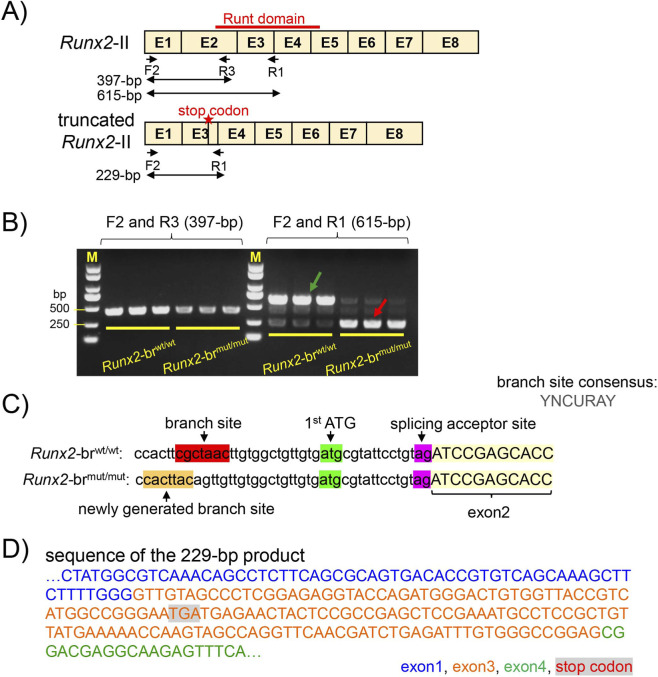
Abnormal RNA splicing from exon 1 to exon 3. **(A)** Schematic representation of wild-type *Runx2*-II and truncated *Runx2*-II, the location of the primers, and the expected size of the PCR products. **(B)** PCR analysis using cDNA from the newborn calvaria of *Runx2*-br^wt/wt^ and *Runx2*-br^mut/mut^ mice. A 397-bp product corresponding to *Runx2*-II was amplified with primers F2 and R3 in both genotypes, but the band intensity in *Runx2*-br^mut/mut^ mice was lower than that in *Runx2*-br^wt/wt^ mice. Using primers F2 and R1, a 615-bp product (green arrow) was detected in *Runx2*-br^wt/wt^ mice, whereas a 229-bp product (red arrow) was detected in *Runx2*-br^mut/mut^ mice. **(C)** Putative branch site. **(D)** Sequence of the 229-bp product.

To investigate how the branch-site mutation affected *Runx2* isoform expression, we performed PCR using cDNA from the calvaria of *Runx2*-br^wt/wt^ and *Runx2*-br^mut/mut^ mice. Forward primer F2, which is located in exon 1, was paired with reverse primer R3 or R1, which are located in exon 2 or exon 3–4, respectively (Figures 1A, 2A). Amplification with F2 and R3 yielded the expected 397-bp product in both genotypes, but the band intensity was weaker in *Runx2*-br^mut/mut^ mice, indicating that intron 1 splicing occurred with reduced efficiency in *Runx2*-br^mut/mut^ mice (Figures 2A,B). Based on the conserved splicing branch site sequence (YNCURAY), we identified a candidate for a newly generated branch site sequence (CACTTAC) 36-bp upstream of the 3′ splicing site of exon 2 (Figure 2C). When using F2 and R1 for PCR, the expected 615-bp product was observed in *Runx2*-br^wt/wt^ mice, whereas a novel 229-bp product appeared in *Runx2*-br^mut/mut^ mice (Figures 2A,B). The sequence of the 229-bp product indicated that exon 1 was spliced to exon 3 (Figures 2B,D). Exon 2 skipping resulted in the appearance of a stop codon in exon 3, and the truncated protein lacked the runt domain (Figures 2A,D).

### 3.2 *Runx2*-II mRNA was severely reduced but *Runx2*-I mRNA was increased in *Runx2*-br^mut/mut^ mice

**FIGURE 3:**
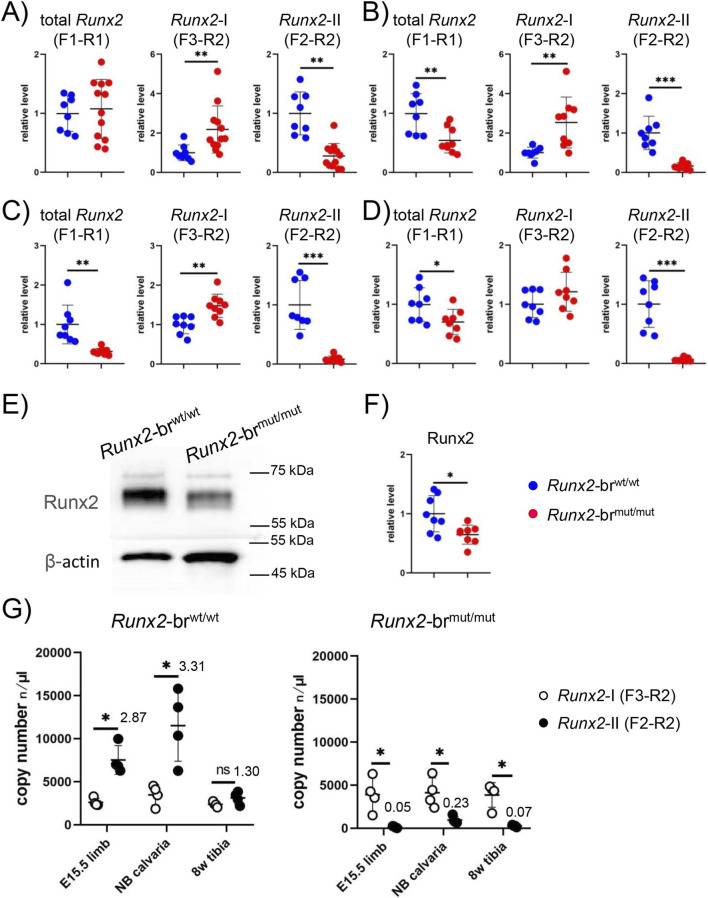
The expression of total *Runx2*, *Runx2*-I, and *Runx2*-II in *Runx2*-br^mut/mut^ mice. **(A–D)** Real-time RT-PCR analysis of total *Runx2*, *Runx2*-I, and *Runx2*-II using RNA isolated from E15.5 limbs **(A)** newborn calvaria **(B)** and limbs **(C)** and 8-week-old tibiae **(D)** of *Runx2*-br^wt/wt^, *Runx2*-br^wt/mut^, and *Runx2*-br^mut/mut^ mice. The values of *Runx2*-br^wt/wt^ mice were defined as 1, and the relative levels are shown. The number of mice analyzed: n = 8 (*Runx2*-br^wt/wt^) and n = 12 (*Runx2*-br^mut/mut^) at E15.5; n = 8 (*Runx2*-br^wt/wt^) and n = 9 (*Runx2*-br^mut/mut^) at newborn, n = 8 (*Runx2*-br^wt/wt^) and n = 8 (*Runx2*-br^mut/mut^) at 8 weeks of age. **(E,F)** Western blot analysis. Proteins were extracted from newborn calvariae, Western blotting was performed using anti-Runx2 antibody, and β-actin was used as an internal control **(E)**. The intensities of bands were normalized against β-actin, normalized values in the average of *Runx2*-br^wt/wt^ mice were set as 1, and relative levels are shown in **(F)**. The number of mice analyzed was n = 8 (*Runx2*-br^wt/wt^) and n = 8 (*Runx2*-br^mut/mut^). *Versus *Runx2*-br^wt/wt^ mice, *p < 0.05, **p < 0.01, ***p < 0.001. **(G)** Droplet digital RT-PCR analysis of *Runx2*-I and *Runx2*-II expression in RNA from E15.5 limbs, newborn calvariae, and 8-week-old tibiae of *Runx2*-br^wt/wt^ and *Runx2*-br^mut/mut^ mice. Four mice were analyzed for each tissue. *Versus *Runx2*-I, *p < 0.05.

To assess isoform-specific expression, we designed primer sets targeting total *Runx2*, *Runx2*-I, and *Runx2*-II (Figure 1A). Total *Runx2* mRNA was amplified using a primer set in exons 2 and 3–4 (F1–R1). *Runx2*-I mRNA was detected using a primer set located upstream and downstream of the splicing site for *Runx2*-II in exon 2 (F3–R2). To detect *Runx2*-II mRNA, spliced mRNA from exons 1 to 2 (F2–R3) was amplified. The levels of total *Runx2* in newborn calvariae and limbs and 8-week-old tibiae of *Runx2*-br^mut/mut^ mice were 54%, 31%, and 70% of those of *Runx2*-br^wt/wt^ mice, respectively, whereas those in the E15.5 limbs were comparable between them (Figures 3A–D). The levels of *Runx2*-I in *Runx2*-br^mut/mut^ mice were higher than those in *Runx2*-br^wt/wt^ mice in all samples, except for the 8-week-old tibiae (Figures 3A–D). The levels of *Runx2*-II in *Runx2*-br^mut/mut^ mice were severely reduced in all samples compared to those in *Runx2*-br^wt/wt^ mice, although the reduction of *Runx2*-II in E15.5 limbs was milder than that in the other samples (Figures 3A–D). Consistent with the mRNA analysis, Runx2 protein levels in the newborn calvariae of *Runx2*-br^mut/mut^ mice were lower than those in *Runx2*-br^wt/wt^ mice (Figures 3E,F).

Droplet digital RT-PCR was performed to assess the relative abundance of *Runx2*-I and *Runx2*-II mRNA in *Runx2*-br^wt/wt^ and *Runx2*-br^mut/mut^ mice. Copy numbers of *Runx2*-II in *Runx2*-br^wt/wt^ mice were approximately three times higher than those of *Runx2*-I in E15.5 limbs and newborn calvariae, whereas those of *Runx2*-I mRNA and *Runx2*-II were similar in 8-week-old tibiae (Figure 3G). In contrast, the copy numbers of *Runx2*-II were extremely lower than those of *Runx2*-I in *Runx2*-br^mut/mut^ mice (Figure 3G).

### 3.3 Delayed ossification in both intramembranous and endochondral bones in *Runx2*-br^mut/mut^ embryos

**FIGURE 4:**
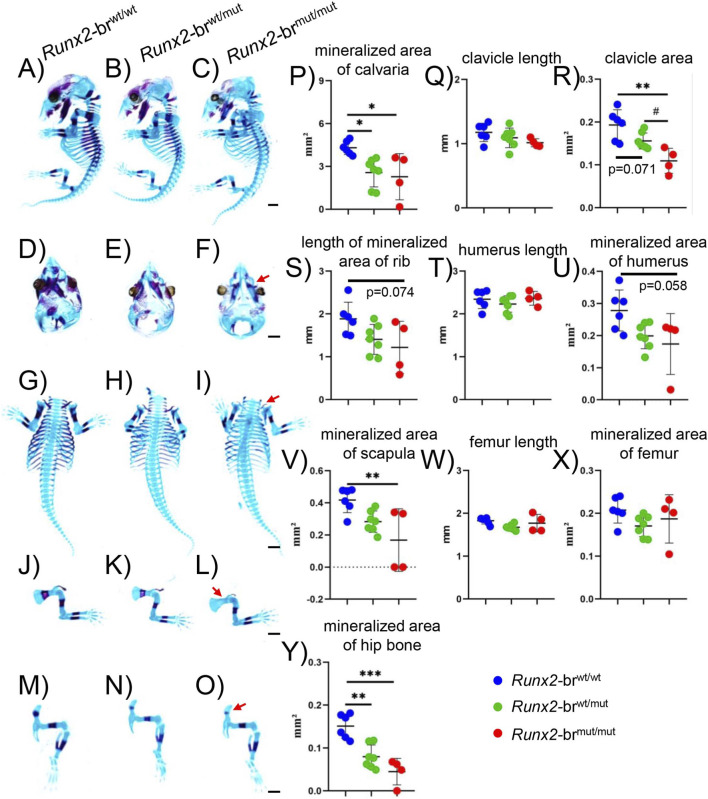
Skeletal system of *Runx2*-br^mut/mut^ embryos at E15.5. **(A–O)** Lateral view of whole skeletons **(A–C)** top view of skulls **(D–F)** clavicle, ribs, and vertebrae **(G–I)** forelimbs **(J–L)** and hindlimbs **(M–O)** in *Runx2*-br^wt/wt^, *Runx2*-br^wt/mut^, and *Runx2*-br^mut/mut^ mice. Red arrows in **(F,I,L,O)** indicate less mineralized frontal bone, clavicle, scapula, and hip bone, respectively, in *Runx2*-br^mut/mut^ mice. Scale bars: 1 mm. **(P–Y)** Quantification of skeletal parameters: mineralized area of calvariae **(P)** clavicle length **(Q)** clavicle area **(R)** length of mineralized area of ribs **(S)** humerus length **(T)** mineralized area of humeri **(U)** mineralized area of scapulae **(V)** femur length **(W)** mineralized area of femurs **(X)** and mineralized area of hip bones **(Y)** in *Runx2*-br^wt/wt^, *Runx2*-br^wt/mut^, and *Runx2*-br^mut/mut^ mice. The number of mice analyzed, n = 6 (*Runx2*-br^wt/wt^), n = 7 (*Runx2*-br^wt/mut^), and n = 4 (*Runx2*-br^mut/mut^). *Versus *Runx2*-br^wt/wt^ mice, ^#^Versus *Runx2*-br^wt/mut^ mice, *^,#^p < 0.05, **p < 0.01, ***p < 0.001.

To evaluate skeletal development, we performed alcian blue and alizarin red staining at E15.5 (Figure 4). Mineralization of both intramembranous and endochondral bones was delayed in *Runx2*-br^mut/mut^ mice compared with *Runx2*-br^wt/wt^ and *Runx2*-br^wt/mut^ mice (Figures 4A–O). Mineralization of the frontal and parietal bones, maxilla, and mandible was delayed in *Runx2*-br^mut/mut^ mice (Figures 4A–F,P). The area, but not the length, of the clavicles in *Runx2*-br^mut/mut^ mice was less than that in *Runx2*-br^wt/wt^ and *Runx2*-br^wt/mut^ mice (Figures 4G–I,Q, R). The mineralization of the scapula and hip bones in *Runx2*-br^mut/mut^ mice was significantly and that of the ribs and humeri was marginally less than that in *Runx2*-br^wt/wt^ mice (Figures 4A–C,G–O,S,U,V,Y). Mineralization of hip bones in *Runx2*-br^wt/mut^ mice was also significantly less than that in *Runx2*-br^wt/wt^ mice, indicating that the heterozygous mutation of the branch site also shows mild phenotypes (Figures 4M,N,Y). The lengths of the humeri and femurs and the mineralized area of the femurs were comparable among the three groups (Figures 4T,W,X).

**FIGURE 5:**
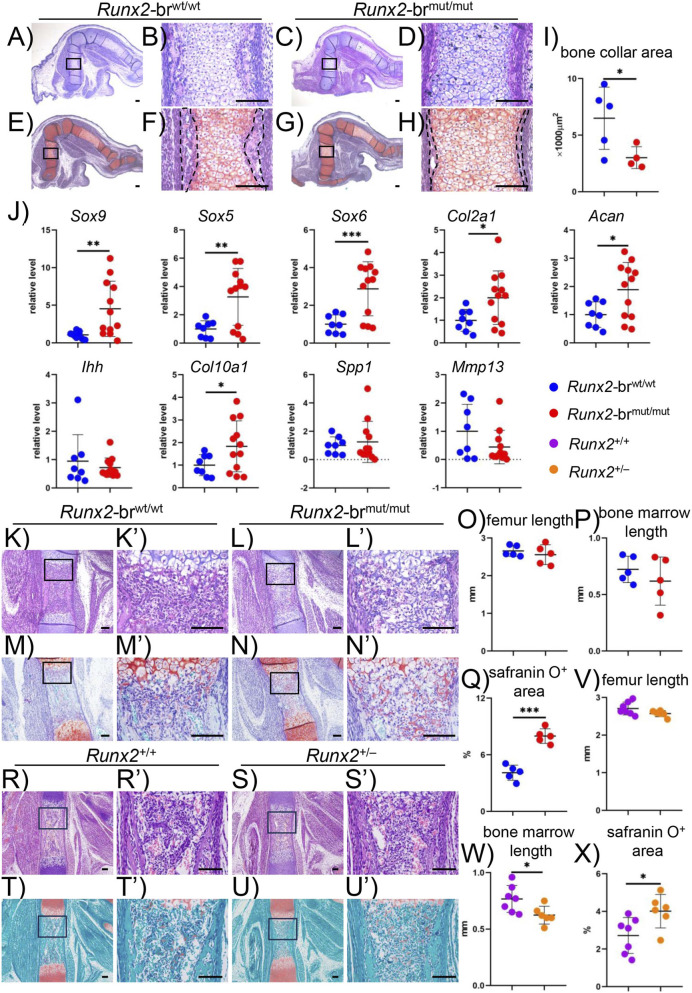
Histological analyses of *Runx2*-br^wt/wt^, *Runx2*-br^mut/mut^, *Runx2* ^+/+^, and *Runx2* ^+/−^ embryos and real‐time RT‐PCR analysis of chondrocyte differentiation markers. **(A–I)** H‐E staining **(A–D)** and Safranin O staining **(E–H)** of femoral sections from *Runx2*-br^wt/wt^ and *Runx2*-br^mut/mut^ embryos at E15.5. The boxed regions are magnified in the right column. Scale bars: 0.1 mm. The bone collar area was measured in **(F,H)** and quantitative data are shown in **(I)**. The number of mice analyzed was n = 5 (*Runx2*-br^wt/wt^) and n = 4 *(Runx2*-br^mut/mut^). **(J)** Real-time RT‐PCR analysis of RNA extracted from hindlimb skeletons of *Runx2*-br^wt/wt^ and *Runx2*-br^mut/mut^ embryos at E15.5. The values in *Runx2*-br^wt/wt^ mice were defined as 1, and the relative levels are shown. The number of mice analyzed: n = 8 (*Runx2*-br^wt/wt^) and n = 12 (*Runx2*-br^mut/mut^). **(K–X)** H‐E **(K,L,R,S)** and Safranin O **(M,N,T,U)** staining, femur length **(O,V)** bone marrow length **(P,W)** and percentage of safranin O-positive area in the bone marrow **(Q,X)** in the femoral sections of *Runx2*-br^wt/wt^ and *Runx2*-br^mut/mut^ mice **(K–Q)** and *Runx2* ^+/+^ and *Runx2* ^+/−^ mice **(R–X)** at E16.5. Scale bars: 0.1 mm. The number of mice analyzed: n = 5 (*Runx2*-br^wt/wt^) and n = 5 (*Runx2*-br^mut/mut^); n = 7 (*Runx2* ^+/+^) and n = 6 (*Runx2* ^+/−^). *Versus *Runx2*-br^wt/wt^ mice or *Runx2* ^+/+^ mice, *p < 0.05, **p < 0.01, ***p < 0.001.

Histological analysis of E15.5 femurs revealed that bone collar formation in *Runx2*-br^mut/mut^ embryos was less than that in *Runx2*-br^wt/wt^ embryos (Figures 5A–I). Real-time RT-PCR analysis of hindlimbs showed upregulation of immature chondrocyte marker genes, including *Sox9*, *Sox5*, *Sox6*, *Col2a1*, *Acan*, and a mature chondrocyte marker gene *Col10a1*, in *Runx2*-br^mut/mut^ embryos compared to that in *Runx2*-br^wt/wt^ embryos. *Spp1* is expressed in terminal hypertrophic chondrocytes and immature osteoblasts, whereas *Mmp13* is mainly expressed in terminal hypertrophic chondrocytes (Qin et al., 2020). The expression levels of *Spp1* and *Mmp13* were comparable in *Runx2*-br^wt/wt^ and *Runx2*-br^mut/mut^ embryos (Figure 5J). Furthermore, histological assessment at E16.5 demonstrated an expanded Safranin O-positive cartilage area in the bone marrow of *Runx2*-br^mut/mut^ femurs showing a delay in endochondral ossification, but the lengths of the femurs and bone marrow were comparable in *Runx2*-br^wt/wt^ and *Runx2*-br^mut/mut^ embryos (Figures 5K–Q).

To compare the process of endochondral ossification in *Runx2*-br^mut/mut^ embryos with that in *Runx2* ^+/−^ embryos, *Runx2* ^+/−^ embryos and their *Runx2* ^+/+^ littermates were examined at E16.5. Although the femur lengths in *Runx2* ^+/−^ embryos were comparable to those in *Runx2* ^+/+^ embryos, the bone marrow lengths in *Runx2* ^+/−^ femurs were shorter than those in *Runx2* ^+/+^ femurs (Figures 5R,S,V,W). The Safranin O-positive area in the bone marrow of *Runx2* ^+/−^ embryos was larger than that in *Runx2* ^+/+^ embryos, but the increase was milder than that in *Runx2*-br^mut/mut^ embryos (Figures 5Q,T,U,X). These findings indicate that the delay in endochondral ossification in *Runx2* ^+/−^ embryos was greater than that in *Runx2*-br^mut/mut^ embryos.

### 3.4 The development of clavicles and limb bones in *Runx2*-br^mut/mut^ newborns was prominently and mildly less affected than that in *Runx2* ^+/−^ newborns, respectively

**FIGURE 6:**
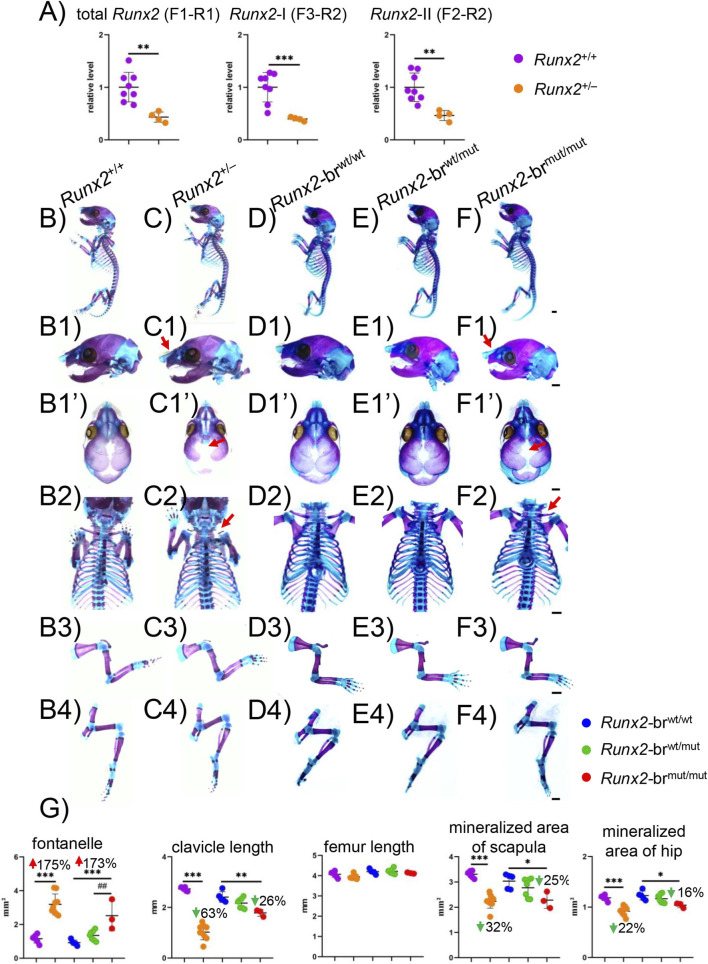
Skeletal system of *Runx2* ^+/−^ and *Runx2*-br^mut/mut^ newborns. **(A)** Real-time RT-PCR was performed using 4-week-old calvaria from *Runx2* ^+/+^ and *Runx2* ^+/−^ mice. The value of *Runx2* ^+/+^ was set to 1, and the relative levels are shown. The number of mice analyzed: n = 8 (*Runx2* ^+/+^), n = 4 (*Runx2* ^+/−^). *Versus *Runx2* ^+/+^ mice, **p < 0.01, ***p < 0.001. **(B–F)** Representative images of the skeletal system: lateral view of whole skeletons **(B–F)** lateral view of skulls **(B1–F1)** top view of skulls **(B1’–F1’)** clavicle, ribs, and sternum **(B2–B2)** hindlimbs **(B3–F3)** and forelimbs **(B4–F4)** in *Runx2* ^+/+^, *Runx2* ^+/−^, *Runx2*-br^wt/wt^, *Runx2*-br^wt/mut^ and *Runx2*-br^mut/mut^ mice. The red arrows in C1, F1, C1′, F1′, C2, and F2 indicate unmineralized nasal bones, open anterior fontanelles, and hypoplastic clavicles in *Runx2* ^+/−^ and *Runx2*-br^mut/mut^ newborns. Scale bars: 1 mm. **(G)** Quantification of the anterior fontanelle area, clavicle length, femur length, and mineralized area of the scapulae and hip bones in *Runx2* ^+/+^, *Runx2* ^+/−^, *Runx2*-br^wt/wt^, *Runx2*-br^wt/mut^, and *Runx2*-br^mut/mut^ mice. The number of mice analyzed: n = 6 (*Runx2* ^+/+^), n = 9 (*Runx2* ^+/−^), n = 5 (*Runx2*-br^wt/wt^), n = 7 (*Runx2*-br^wt/mut^), and n = 3 (*Runx2*-br^mut/mut^). *Versus *Runx2-*br^wt/wt^ mice or *Runx* ^+/+^ mice, #Versus *Runx2-*br^wt/mut^. *p < 0.05, **,##p < 0.01, ***p < 0.001.

*Runx2* ^+/−^ mice, in which the expression levels of total *Runx2*, *Runx2*-I, and *Runx2*-II were approximately half those in *Runx2* ^+/+^ mice (Figure 6A), displayed the CCD phenotype. Therefore, we compared the skeletal development of *Runx2* ^+/−^, *Runx2*-br^wt/mut^, and *Runx2*-br^mut/mut^ newborns with their respective controls. Whole-body skeletal size was comparable across all genotypes (Figures 6B–F), and skeletal development in *Runx2*-br^wt/mut^ mice was indistinguishable from that of the *Runx2*-br^wt/wt^ controls. Notably, nasal bone mineralization was absent in both *Runx2* ^+/−^ and *Runx2*-br^mut/mut^ mice (Figure 6C1,F1). Although the anterior fontanelles were opened similarly in both *Runx2* ^+/−^ and *Runx2*-br^mut/mut^ mice (Figures 6B1’–F1’, G), hypoplasia of the clavicles was more severe in *Runx2* ^+/−^ mice (Figures 6B2–F2,G). Although the lengths of the femurs were comparable among all genotypes, the reduction in the mineralized area of the scapula and hip bones was more severe in *Runx2* ^+/−^ mice than in *Runx2*-br^mut/mut^ mice compared to the respective control mice, showing less affected endochondral ossification in *Runx2*-br^mut/mut^ mice than in *Runx2* ^+/−^ mice, as observed in embryos (Figures 6B3–F4,G).

**FIGURE 7:**
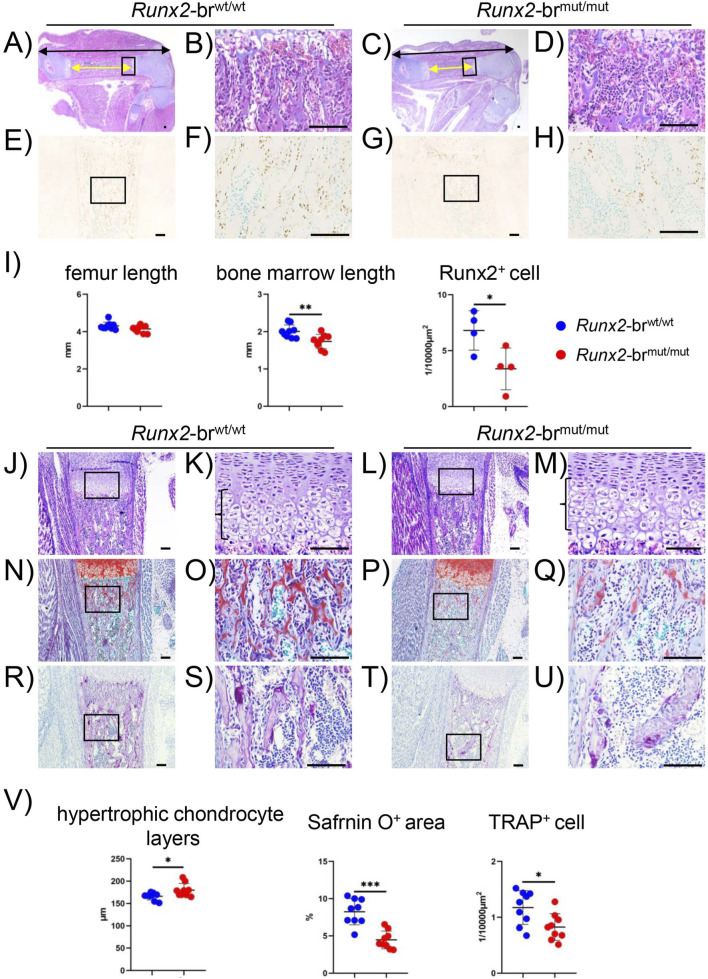
Histological analysis of *Runx2*-br^wt/wt^ and *Runx2*-br^mut/mut^ newborns. **(A–H)** H-E staining **(A–D)** and immunohistochemical analysis using anti-Runx2 antibody **(E–H)** of femoral sections from *Runx2*-br^wt/wt^ and *Runx2*-br^mut/mut^ mice. The boxed regions are magnified in the right column. Scale bars: 0.1 mm. **(I)** Quantification of femur length, black bidirectional arrow line in **(A,C)**, length of bone marrow, yellow bidirectional arrow line in **(A,C)** and Runx2-positive cells in the primary spongiosa. The number of mice analyzed: n = 9 in H-E staining, n = 4 in Runx2 immunostaining. *Versus *Runx2* ^+/+^ or *Runx2*-br^wt/wt^ mice, ^#^Versus *Runx2*-br^wt/mut^ mice. *^,#^p < 0.05, **p < 0.01, ***p < 0.001. **(J–M)** H-E staining. **(N–Q)** Safranin O staining. **(R–U)** TRAP staining. The boxed regions are magnified in the right column. Scale bars: 0.1 mm. **(V)** The lengths of the hypertrophic chondrocyte layers are shown in **(K,M)** the percentages of Safranin O-positive area in the bone marrow were measured using **(O,Q)** and the number of TRAP-positive cells in the bone marrow was counted using **(S,U)**. Nine mice were analyzed for each genotype. *Versus *Runx2*-br^wt/wt^ mice, *p < 0.05, ***p < 0.001.

In the newborn stage, *Runx2*-br^mut/mut^ mice exhibited shorter bone marrow, elongated hypertrophic chondrocyte zones, and less primary spongiosa formation than *Runx2*-br^wt/wt^ mice (Figures 7A–D,I–M,V). Additionally, Runx2^+^ cells, Safranin O^+^ cartilaginous matrix, and TRAP^+^ osteoclasts in the bone marrow of *Runx2*-br^mut/mut^ mice were significantly fewer than those in *Runx2*-br^wt/wt^ mice (Figures 7E–I,N–V). The decrease in the Safranin O^+^ matrix in *Runx2*-br^mut/mut^ mice was likely caused by reduced primary spongiosa formation. Combined with the data from E15.5 and E16.5 (Figure 5), these findings indicate that endochondral ossification was delayed and osteoblast differentiation was impaired in *Runx2*-br^mut/mut^ mice. Moreover, the impaired formation of primary spongiosa with the reduced Runx2^+^ osteoblasts suggests that the transdifferentiation of terminal hypertrophic chondrocytes into osteoblasts was impaired.

### 3.5 Impaired bone development in *Runx2*-br^mut/mut^ mice at 8 weeks of age

**FIGURE 8:**
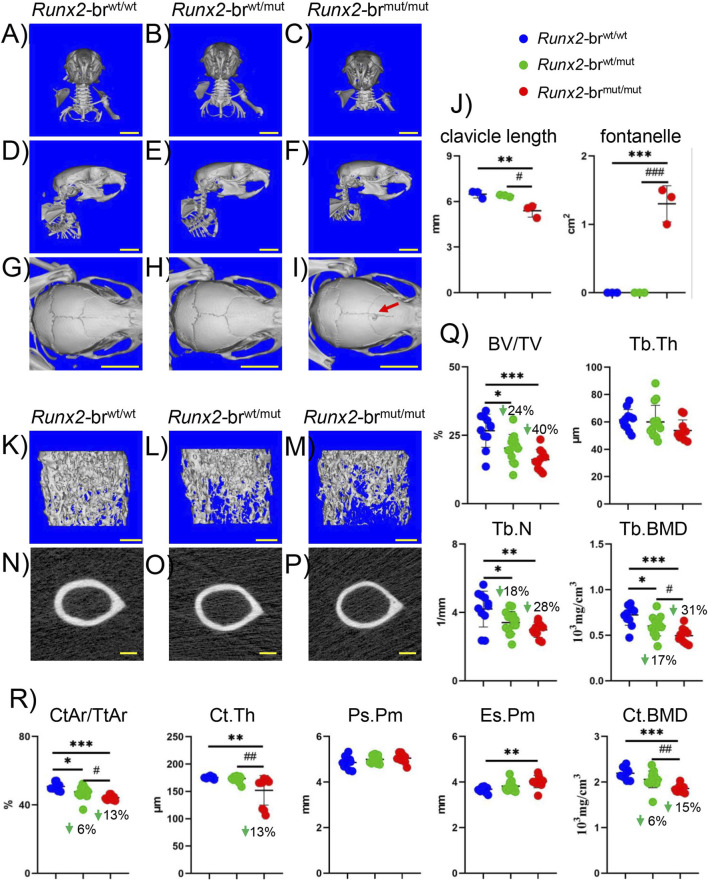
Micro-CT analyses of *Runx2*-br^wt/wt^, *Runx2*-br^wt/mut^, and *Runx2*-br^mut/mut^ mice at 8 weeks of age. **(A–I)** Micro-CT images of the head and neck. Frontal view **(A–C)** lateral view **(D–F)** and top view **(G–I)** of the skulls. The right clavicle was cut to detach the humerus, whereas the left clavicle remained intact in the frontal view. The red arrow indicates the open frontal sutures **(I)**. Scale bars: 5 mm. **(J)** Clavicle length and unmineralized area of the fontanel. n = 3. **(K–M)** Three-dimensional trabecular bone architecture of the distal femoral metaphysis. **(N–P)** Micro-CT images of the cortical bone at the mid-diaphysis of the femurs. **(Q)** Bone volume (BV)/tissue volume (TV), trabecular thickness (Tb.Th), trabecular number (Tb.N), and trabecular bone mineral density (Tb.BMD) in male mice. **(R)** Cortical area (CtAr)/total area (TtAr), cortical thickness (Ct.Th), periosteal perimeter (Ps.Pm), endosteal perimeter (Es.Pm), and cortical BMD (Ct.BMD) in male mice. Scale bars: 0.5 mm. The number of mice analyzed was n = 11 (*Runx2*-br^wt/wt^), n = 14 (*Runx2*-br^wt/mut^), and n = 10 (*Runx2*-br^mut/mut^). *Versus *Runx2*-br^wt/wt^ mice, ^#^Versus *Runx2*-br^wt/mut^ mice, *^,#^p < 0.05, **^,##^p < 0.01, ***^,###^p < 0.001.

Skeletal analysis using micro-CT revealed that incisor alignment was normal, clavicle length was slightly shorter, and all sutures, except the posterior frontal suture, were closed in *Runx2*-br^mut/mut^ mice at 8 weeks of age (Figures 8A–J). The trabecular bone volume, trabecular number, trabecular bone mineral density (BMD), and cortical area in *Runx2*-br^wt/mut^ and *Runx2*-br^mut/mut^ mice were lower than those in *Runx2*-br^wt/wt^ mice (Figures 8K–R). The cortical thickness and BMD were lower, and the endosteal perimeter was greater in *Runx2*-br^mut/mut^ mice than in *Runx2*-br^wt/wt^ mice (Figures 8N–P,R). The reductions in BMD in the trabecular and cortical bones were 6% and 4%, respectively, in *Runx2* ^+/−^ femurs at 10 weeks of age (Jiang et al., 2022), whereas they were 31% and 15%, respectively, in *Runx2*-br^mut/mut^ mice compared with those in the respective control mice. Thus, the reduction in BMD was more prominent in *Runx2*-br^mut/mut^ mice.

**FIGURE 9:**
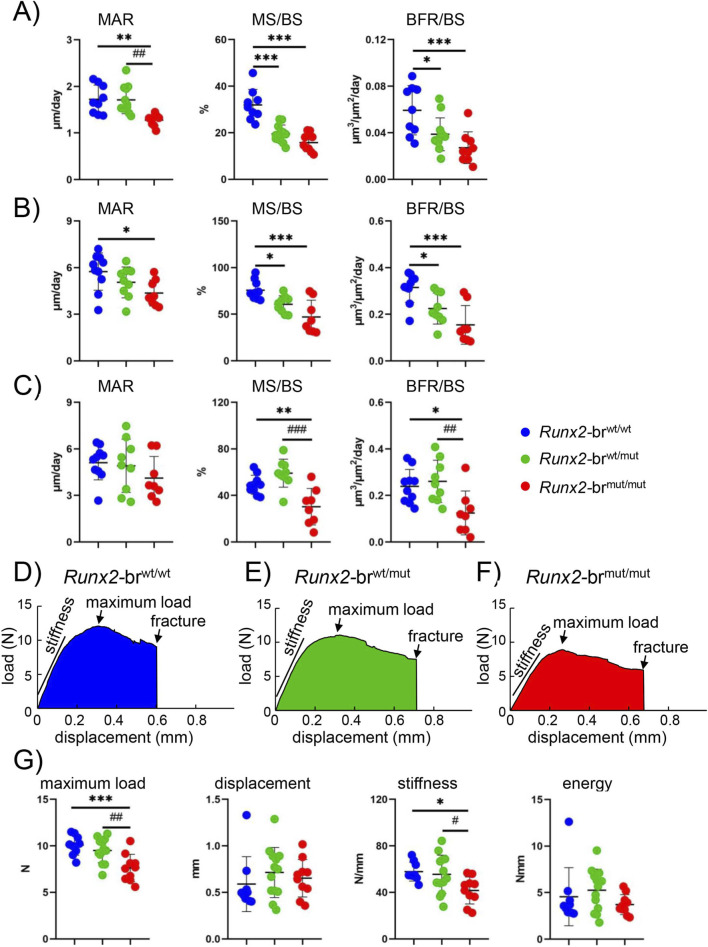
Dynamic bone histomorphometric analyses of femoral trabecular and cortical bone and three-point bending test using humeri in male *Runx2*-br^wt/wt^, *Runx2*-br^wt/mut^, and *Runx2*-br^mut/mut^ mice at 8 weeks of age. **(A–C)** Bone histomorphometric analysis. Mineral apposition rate (MAR), mineralizing surface (MS/BS), and bone formation rate (BFR/BS) in the trabecular bone **(A)** periosteum **(B)** and endosteum **(C)** of *Runx2*-br^wt/wt^, *Runx2*-br^wt/mut^, and *Runx2*-br^mut/mut^ mice. The number of mice analyzed: n = 9 (*Runx2*-br^wt/wt^), n = 12 (*Runx2*-br^wt/mut^), and n = 9 (*Runx2*-br^mut/mut^) in trabecular bone; n = 10 (*Runx2*-br^wt/wt^), n = 9 (*Runx2*-br^wt/mut^), and n = 8 (*Runx2*-br^mut/mut^) in cortical bone. **(D–G)** Three-point bending tests. Representative load-displacement curves of the humeri in *Runx2*-br^wt/wt^ **(D)** *Runx2*-br^wt/mut^ **(E)** and *Runx2*-br^mut/mut^ **(F)** mice and the mechanical parameters **(G)**. The number of mice analyzed: n = 9 (*Runx2*-br^wt/wt^), n = 13 (*Runx2*-br^wt/mut^), and n = 10 (*Runx2*-br^mut/mut^). *Versus *Runx2*-br^wt/wt^ mice, ^#^versus *Runx2*-br^wt/mut^ mice, *^,#^p < 0.05, **^,##^p < 0.01, ***^,###^p < 0.001.

Dynamic bone histomorphometric analyses of trabecular bone in femurs revealed that the mineral apposition rate (MAR), mineralizing surface (MS/BS), and bone formation rate (BFR/BS) were lower in *Runx2*-br^mut/mut^ mice, and the MS/BS and BFR/BS were also lower in *Runx2*-br^wt/mut^ mice than in *Runx2*-br^wt/wt^ mice (Figure 9A). Dynamic bone histomorphometric analyses of the cortical bone in the periosteum of the femurs showed that the MAR, MS/BS, and BFR in *Runx2*-br^mut/mut^ mice were lower, and the MS/BS and BFR in *Runx2*-br^wt/mut^ mice were lower than those in *Runx2*-br^wt/wt^ mice (Figure 9B). In the endosteum of the femurs, MS/BS and BFR/BS in *Runx2*-br^mut/mut^ mice were lower than those in *Runx2*-br^wt/mut^ and *Runx2*-br^wt/wt^ mice (Figure 9C).

Mechanical testing by three-point bending of the humeri revealed compromised bone strength in *Runx2*-br^mut/mut^ mice. The maximum load and stiffness of the humeri in *Runx2*-br^mut/mut^ mice were lower than those in *Runx2*-br^wt/wt^ and *Runx2*-br^wt/mut^ mice, whereas the displacement and energy to failure were comparable among the three groups (Figures 9D–G). These comprehensive analyses demonstrated that *Runx2*-br^mut/mut^ mice exhibit substantial defects in both bone formation capacity and mechanical integrity.

### 3.6 Impaired osteoblast differentiation in *Runx2*-br^mut/mut^ mice

**FIGURE 10:**
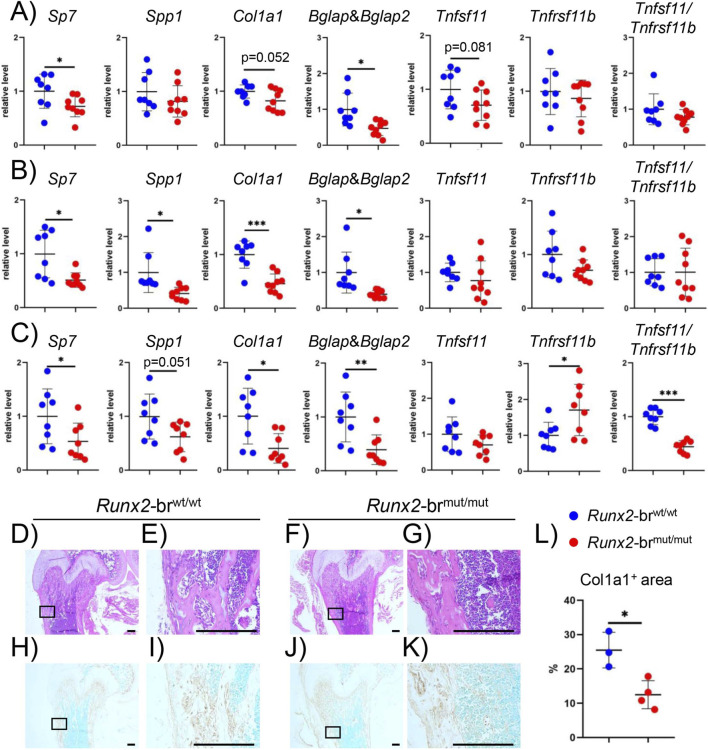
Real-time RT-PCR and immunohistochemical analyses. **(A–C)** Real-time RT-PCR using RNA from newborn calvariae **(A)** and limbs **(B)** and 8-week-old tibiae **(C)** of *Runx2*-br^wt/wt^ and *Runx2*-br^mut/mut^ mice. The values of *Runx2*-br^wt/wt^ mice were defined as 1, and the relative levels are shown. The number of mice analyzed: n = 8 (*Runx2*-br^wt/wt^) and n = 9 (*Runx2*-br^mut/mut^) in newborns; n = 8 in 8-week-old mice. **(D–K)** H&E staining **(D–G)** and immunohistochemical analysis of Col1a1 **(H–K)** using the femurs of *Runx2*-br^wt/wt^ **(D,H)** and *Runx2*-br^mut/mut^ **(F,J)** mice at 3 weeks of age. The boxed regions in D, F, H, and J were magnified in **(E,G,I,K)** respectively. **(L)** The percentages of Col1a1-positive area/bone area. Scale bars = 0.1 mm. The number of mice analyzed: n = 3 (*Runx2*-br^wt/wt^) and n = 4 (*Runx2*-br^mut/mut^). *Versus *Runx2*-br^wt/wt^ mice, *p < 0.05, **p < 0.01, ***p < 0.001.

Real-time RT-PCR analysis of osteoblast marker genes, including *Sp7* and *Col1a1* for immature and mature osteoblasts with an increase of *Col1a1* during osteoblast maturation, *Spp1* for immature osteoblasts, *Bglap/Bglap2* for mature osteoblasts, and osteoclastogenesis-related genes, including *Tnfsf11* (*Rankl*) and *Tnfrsf11b* (*Opg*), was performed using newborn calvaria and hindlimbs and 8-week-old tibiae (Figures 10A–C). The expression of *Sp7* and *Bglap/Bglap2*, but not *Spp1*, *Col1a1*, *Tnfsf11*, and *Tnfrsf11b*, in the calvariae of *Runx2*-br^mut/mut^ newborns was lower than that in *Runx2*-br^wt/wt^ newborns, and the ratios of *Tnfsf11* and *Tnfrsf11b* were comparable between them (Figure 10A). In the hindlimbs of newborns, the expression of *Sp7*, *Spp1*, *Col1a1*, and *Bglap/Bglap2* in *Runx2*-br^mut/mut^ mice was lower than that in *Runx2*-br^wt/wt^ mice, whereas the expression of *Tnfsf11* and *Tnfrsf11b* and the ratios of *Tnfsf11* and *Tnfrsf11b* were comparable between the two groups (Figure 10B). In 8-week-old tibiae, the expression of *Sp7*, *Col1a1*, and *Bglap/Bglap2* was lower, that of *Tnfrsf11b* was higher, and the ratios of *Tnfsf11* and *Tnfrsf11b* were lower in *Runx2*-br^mut/mut^ mice than in *Runx2*-br^wt/wt^ mice (Figure 10C). These results indicate that osteoblast differentiation was inhibited and osteoclastogenesis was likely to be suppressed in the adult limbs of *Runx2*-br^mut/mut^ mice. The protein level of Col1a1 was examined using the 3-week-old femurs by immunohistochemistry. The Col1a1-positive area in *Runx2*-br^mut/mut^ mice were lower than that in *Runx2*-br^wt/wt^ mice (Figures 10D–L).

### 3.7 The proliferation of suture mesenchymal cells was increased in *Runx2*-br^mut/mut^ mice

**FIGURE 11:**
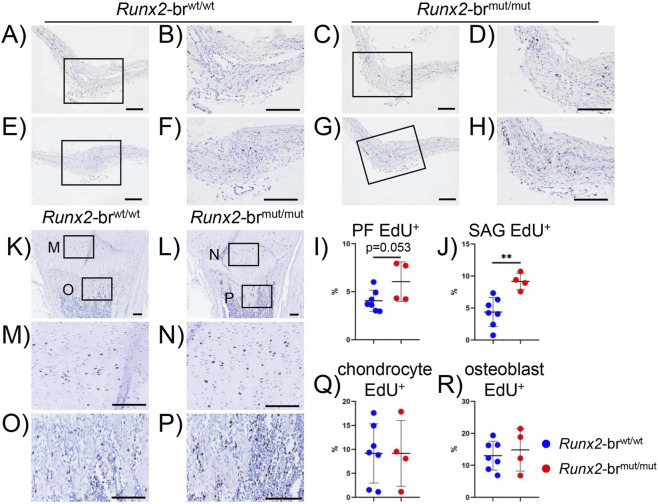
EdU staining of suture mesenchymal cells and femurs in *Runx2*-br^wt/wt^ and *Runx2*-br^mut/mut^ mice at P7. **(A–J)** EdU staining of the posterior frontal (PF) suture **(A,C)** and sagittal (SAG) suture **(E,G)** at P7. The boxed regions are magnified in the right column. The number of EdU^+^ cells and the total number of cells in the PF suture **(B,D)** and SAG suture **(F,H)** were counted, and the percentages of EdU^+^ cells are shown in I and J, respectively. **(K–P)** EdU staining in femoral sections from *Runx2*-br^wt/wt^ and *Runx2*-br^mut/mut^ mice. The upper boxed regions (proliferating chondrocyte layer) and the lower boxed regions (primary spongiosa) in **(K,L)** are magnified in **(M–P)** respectively. Scale bars: 0.1 mm. **(Q)** Percentage of EdU^+^ chondrocytes. **(R)** Percentage of EdU^+^ osteoblasts. Scale bars: 0.1 mm. The number of mice analyzed: n = 5 (*Runx2*-br^wt/wt^) and 4 (*Runx2*-br^mut/mut^). *Versus *Runx2*-br^wt/wt^ mice, *p < 0.05, **p < 0.01, ***p < 0.001.

Although *Runx2* ^+/−^ mice showed widely open fontanelles at 14 weeks of age (Jiang et al., 2022), all fontanelles were mostly closed in *Runx2*-br^mut/mut^ mice at 8 weeks of age (Figures 8G–J). As both *Runx2*-br^mut/mut^ and *Runx2* ^+/−^ mice expressed half the amount of *Runx2* mRNA of wild-type mice in calvariae (Figures 3B, 6A), we examined the mechanism of the less affected calvarial development in *Runx2*-br^mut/mut^ mice compared with *Runx2* ^+/−^ mice. We previously reported that reduced proliferation of suture mesenchymal cells is one of the causes of open fontanelle in *Runx2* ^+/−^ mice (Qin et al., 2019). Thus, we examined the proliferation of suture mesenchymal cells in *Runx2*-br^mut/mut^ mice at P7. The frequencies of EdU^+^ mesenchymal cells increased marginally in the posterior frontal (PF) suture and significantly in the sagittal (SAG) suture in *Runx2*-br^mut/mut^ mice compared to those in *Runx2*-br^wt/wt^ mice (Figures 11A–J). In contrast, the frequencies of EdU^+^ chondrocytes in the proliferating layers of the growth plate and osteoblasts in the trabecular bone in the femurs were comparable in *Runx2*-br^wt/wt^ and *Runx2*-br^mut/mut^ mice at P7 (Figures 11K–R). These findings suggest that the enhanced mesenchymal cell proliferation in the sutures may have contributed to the less affected calvarial development in *Runx2*-br^mut/mut^ mice compared to *Runx2* ^+/−^ mice.

### 3.8 Impaired commitment to an osteoblast lineage in BMSCs and impaired maturation of POB in *Runx2*-br^mut/mut^ mice

**FIGURE 12:**
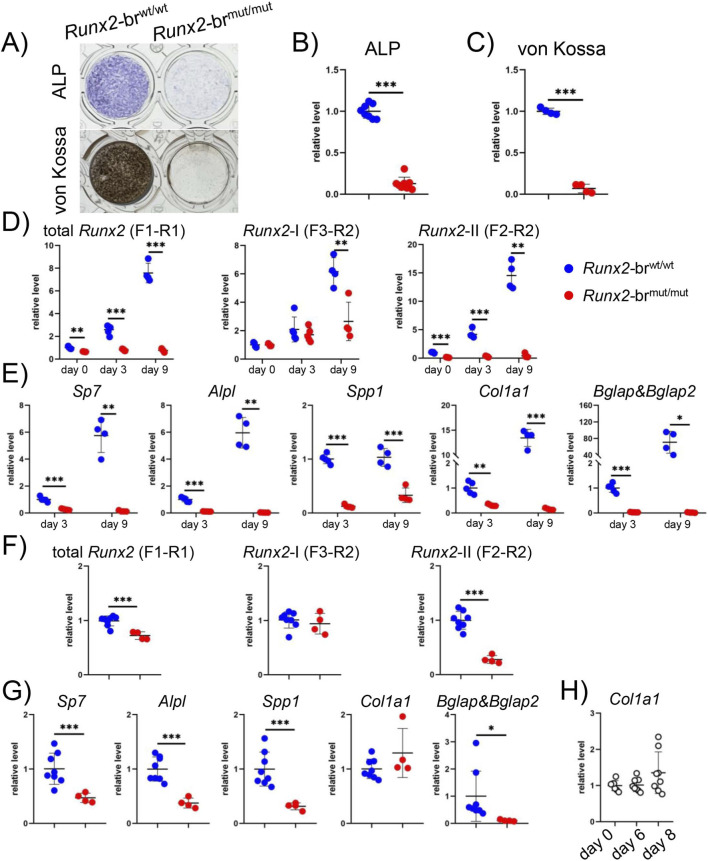
Culture of BMSCs and POB. **(A–C)** ALP and von Kossa staining of BMSCs from *Runx2*-br^wt/wt^ and *Runx2*-br^mut/mut^ mice. **(D–G)** Real-time RT-PCR analysis. Total *Runx2*, *Runx2*-I, and *Runx2*-II expression at days 0, 3, and 9 in BMSCs **(D)** and at day 6 in POB **(F)** and osteoblast differentiation marker gene expression at days 3 and 9 in BMSCs **(E)** and at day 6 in POB **(G)** were examined. The values of *Runx2*-br^wt/wt^ mice were defined as 1, and the relative levels are shown. **(H)** Col1a1 expression in *Runx2*-br^wt/wt^ POB during culture. The values of day 0 were defined as 1, and the relative levels are shown. Similar results were obtained in three independent experiments, and representative data are shown. The number of mice analyzed was n = 8 (*Runx2*-br^wt/wt^) and n = 8 (*Runx2*-br^mut/mut^) in ALP staining, n = 4 (*Runx2*-br^wt/wt^) and n = 4 (*Runx2*-br^mut/mut^) in von Kossa staining, n = 4 (*Runx2*-br^wt/wt^) and n = 4 (*Runx2*-br^mut/mut^) in day 0 and day 9 BMSCs differentiation, n = 5 (*Runx2*-br^wt/wt^) and n = 5 (*Runx2*-br^mut/mut^) in day 3 BMSCs differentiation, n = 8 (*Runx2*-br^wt/wt^) and n = 4 (*Runx2*-br^mut/mut^) in F and G, n = 7, n = 8 and n = 8 in day 0, day 6 and day 8 of POB differentiation (H), respectively. *Versus *Runx2*-br^wt/wt^ mice, *p < 0.05, **p < 0.01, ***p < 0.001.

We compared the osteoblast differentiation of BMSCs from *Runx2*-br^wt/wt^ and *Runx2*-br^mut/mut^ mice. The levels of ALP and von Kossa staining, which represent osteoblast differentiation at the early and late stages, respectively, were drastically reduced in *Runx2*-br^mut/mut^ mice compared to those in *Runx2*-br^wt/wt^ mice (Figures 12A–C). Real-time RT-PCR was performed using BMSCs at confluence (day 0) and 3 and 9 days after confluence. All *Runx2* mRNAs, including total *Runx2*, *Runx2*-I, and *Runx2*-II, markedly increased during the culture of *Runx2*-br^wt/wt^ BMSCs. In contrast, the increase during culture was virtually absent in total *Runx2* and *Runx2*-II and very mild in *Runx2*-I in *Runx2*-br^mut/mut^ BMSCs (Figure 12D). The expression of *Sp7*, *Alpl*, *Spp1*, *Col1a1* and *Bglap/Bglap2* in *Runx2*-br^mut/mut^ BMSCs was severely reduced at all examined points as compared with those in *Runx2*-br^wt/wt^ BMSCs, and the expressions except *Spp1* in *Runx2*-br^mut/mut^ BMSCs were virtually absent at day 9 (Figure 12E). These findings indicate that *Runx2*-II is required for BMSCs commitment to the osteoblast lineage.

Real-time RT-PCR was performed using POB 6 days after confluence. Total *Runx2* and *Runx2*-II, but not *Runx2*-I, levels were reduced in *Runx2*-br^mut/mut^ POB compared to those in *Runx2*-br^wt/wt^ POB (Figure 12F). The expression levels of *Sp7*, *Alpl*, *Spp1*, and *Bglap/Bglap2*, but not *Col1a1*, were lower in *Runx2*-br^mut/mut^ POB than in *Runx2*-br^wt/wt^ POB (Figure 12G). As *Col1a1* expression was not upregulated during the culture of *Runx2*-br^wt/wt^ POB (Figure 12H), *Col1a1* was not a marker of mature osteoblasts in POB. These findings indicate that *Runx2*-II is required for the differentiation of committed osteoblasts *in vitro*.

### 3.9 Overexpression of *Runx2* failed to affect endogenous *Runx2* expression and si*Runx2* failed to affect P2 reporter activity

**FIGURE 13:**
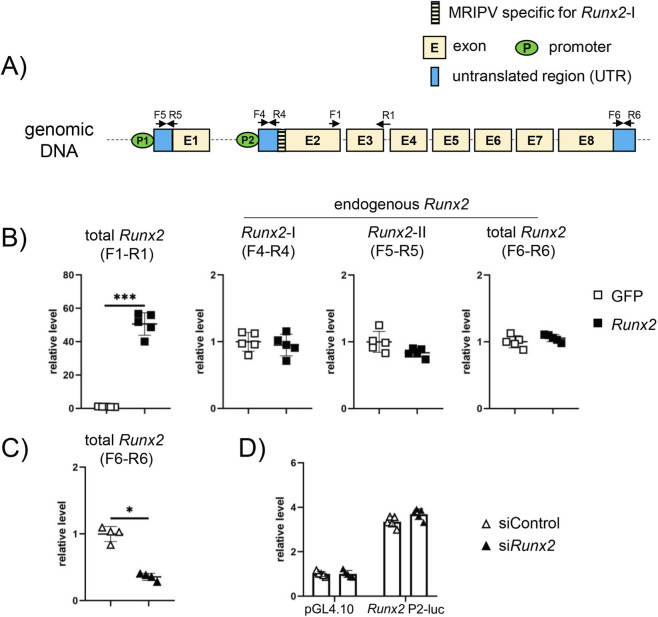
Overexpression of *Runx2* and P2 reporter assay using MC3T3-E1 cells. **(A)** Schematic presentation of untranslated regions of *Runx2* mRNA. Primer sets F1–R1, F4–R4, F5–R5, and F6–R6 were used to detect total Runx2, including exogenous and endogenous *Runx2*, endogenous *Runx2*-I, endogenous *Runx2*-II, and total endogenous *Runx2*, respectively. The R1 primer sequence includes exons 3 and 4. **(B)** Overexpression of *Runx2* in MC3T3-E1 cells. GFP- or *Runx2*-expressing vectors were transfected into MC3T3-E1 cells, and total *Runx2* (F1–R1), including exogenous and endogenous *Runx2*, and endogenous *Runx2*-I (F4–R4), *Runx2*-II (F5–R5), and total *Runx2* (F6–R6) were examined by real-time RT-PCR. **(C,D)** P2 reporter assays. MC3T3-E1 cells were transfected with siRNA for control (siControl) or *Runx2* (si*Runx2*). *Runx2* mRNA was examined 48 h after transfection **(C)**. The value in siControl was defined as 1, and the relative level is shown. ∗Versus siControl, ∗p < 0.05. The transfected cells were further transfected with pGL4.10 empty vector or 1.9-kb P2-Luc, and luciferase activity was measured **(D)**. Similar results were obtained in three independent experiments, and representative data are shown.

To verify whether the upregulation of *Runx2*-I in *Runx2*-br^mut/mut^ mice was due to negative feedback regulation caused by *Runx2* reduction, we overexpressed *Runx2* in osteoblastic MC3T3-E1 cells. Total *Runx2* (F1–R1), including exogenous and endogenous *Runx2*, was upregulated more than 50 times by transfection with the *Runx2* expression vector compared to that with the GFP expression vector. However, the expression of endogenous *Runx2*-I, *Runx2*-II, and total *Runx2*, which were detected using the primer pairs (F4–R4), (F5–R5), and (F6–R6), respectively (Figure 13A), were similar between the GFP and *Runx2* transfected cells (Figure 13B). Furthermore, knockdown of *Runx2* using siRNA in MC3T3-E1 cells had no effect on 1.9-kb P2 reporter activity (Figures 13C,D). These findings indicate that the upregulation of *Runx2*-I in *Runx2*-br^mut/mut^ mice is not due to a negative feedback effect of Runx2 reduction and that P2 activity is not autoregulated by Runx2.

## 4 Discussion

*Runx2*-br^mut/mut^ mice exhibited that Runx2-I and Runx2-II exert different predominant functions in the process of bone development, although they have overlapping functions in intramembranous and endochondral ossification. Runx2-II predominantly functioned in osteoblast differentiation including the commitment to the osteoblast lineage, whereas Runx2-I predominantly functioned in the proliferation of suture mesenchymal cells and greatly contributed to the development of the calvaria and clavicles.

Runx2 protein is weakly expressed in proliferating chondrocytes and strongly expressed in prehypertrophic, hypertrophic, and terminal hypertrophic chondrocytes and osteoblasts in the femur (Matsuo et al., 2025). The expression patterns of the two isoforms were examined by *in situ* hybridization and *Runx2*-II^lacz/lacz^ mice, in which exon 1 was replaced with lacz (Liu et al., 2011; Park et al., 2001). These findings indicate that the expression of *Runx2*-I and *Runx2*-II likely overlaps in preosteoblasts, osteoblasts, prehypertrophic, hypertrophic, and terminal hypertrophic chondrocytes, and the perichondrium/periosteum, whereas *Runx2*-I, but not *Runx2*-II, is expressed in suture mesenchymal cells.

*Runx2*-II^−/−^ mice, in which P1 and exon 1 were deleted, showed severe osteopenia, and most of the mice died neonatally, with less than 10% of the mice surviving for over 6 weeks (Xiao et al., 2004). However, both endochondral and intramembranous bones were formed, indicating the importance of Runx2-I. *Runx2*-I mRNA was upregulated to twice that of wild-type levels. In contrast to *Runx2*-br^mut/mut^ mice, the development of the calvaria and clavicles in *Runx2*-II^−/−^ mice was more severely impaired than that in *Runx2* ^+/−^ mice, although both *Runx2*-br^mut/mut^ and *Runx2*-II^−/−^ mice expressed *Runx2*-I twice that of wild-type mice (Xiao et al., 2004; Zhang et al., 2009). The reduction of *Runx2*-II in *Runx2*-br^mut/mut^ mice was much more severe than that in *Runx2* ^+/−^ mice (Figures 3A–D; Figure 6A). However, the upregulated *Runx2*-I in *Runx2*-br^mut/mut^ mice compensated for the *Runx2*-II reduction in calvaria and clavicle development (Figures 4, 6, 8). The reduction in *Spp1* and *Col1a1* expression in the newborn limbs and 8-week-old tibiae of *Runx2*-br^mut/mut^ mice was not observed in the newborn calvaria (Figures 10A–C), indicating the compensation by Runx2-I in calvaria development. As the reduction of suture mesenchymal cell proliferation is one of the causes of impaired calvaria development in *Runx2* ^+/−^ mice and *Runx2*-I is specifically expressed in suture mesenchymal cells (Qin et al., 2019; Park et al., 2001), it is likely that the upregulated *Runx2*-I in *Runx2*-br^mut/mut^ mice contributed to calvaria development by increasing the proliferation of suture mesenchymal cells. As compensation by *Runx2*-I in the development of calvaria and clavicles was not observed in *Runx2*-II^−/−^ mice (Zhang et al., 2009), a low amount of *Runx2*-II is required for compensation by *Runx2*-I.

Although the levels of total *Runx2* in *Runx2*-br^mut/mut^ limbs were comparable to those in wild-type limbs at E15.5, endochondral ossification was delayed in *Runx2*-br^mut/mut^ mice (Figures 3A, 4, 5). However, the delay in endochondral ossification in *Runx2*-br^mut/mut^ mice was milder than that in *Runx2* ^+/−^ mice, although the total *Runx2* level in the newborn limbs was approximately one-third of that in *Runx2*-br^wt/wt^ mice (Figures 3C, 6) (Jiang et al., 2022). Therefore, these findings indicated that both Runx2-I and Runx2-II contribute to endochondral ossification and Runx2-I can compensate for Runx2-II. In contrast, the delay was much more severe in *Runx2*-II^−/−^ mice than in *Runx2* ^+/−^ mice (Zhang et al., 2009). Therefore, upregulated *Runx2*-I compensated for *Runx2*-II reduction in endochondral ossification in the presence of a small amount of *Runx2*-II. Thus, the small amount of Runx2-II was likely to enhance the contribution of Runx2-I, suggesting the basal requirement of Runx2-II for bone development and dose-independent contribution of the two isoforms, although Runx2 functions have been reported to be dependent on total *Runx2* dosage (Zhang et al., 2009).

The reductions in BMDs in the trabecular and cortical bones of *Runx2*-br^mut/mut^ mice were much more severe than those in *Runx2* ^+/−^ mice, and the expression of *Sp7*, *Col1a1*, and *Bglap/Bglap2* was reduced in *Runx2*-br^mut/mut^ mice but not in *Runx2* ^+/−^ mice (Figures 8, 10C) (Jiang et al., 2022). Severe reductions in BMD and the expression of these genes were also observed in *Runx2*-II^−/−^ mice (Xiao et al., 2005). Therefore, Runx2-II is required for osteoblast differentiation, and Runx2-I cannot compensate for Runx2-II reduction in osteoblast differentiation. Overexpression of either *Runx2*-I or *Runx2*-II in chondrocytes and osteoblasts did not show phenotypical differences in mice, although the dependency on Cbfb was different (Ueta et al., 2001; Kanatani et al., 2006). However, overexpression of *Runx2*-II showed stronger phenotypes than that of *Runx2*-I (Ueta et al., 2001; Kanatani et al., 2006). Therefore, Runx2-II may be more potent than Runx2-I in the formation of a transcriptome or enhanceosome by interacting with other transcription factors and cofactors. As *Tnfrsf11b* expression increased in 8-week-old tibiae (Figure 10C), Runx2-II may play a major role in the negative regulation of *Tnfrsf11b* expression in adult mice, because Runx2 inhibits *Tnfrsf11b* expression (Enomoto et al., 2003).

*Runx2*-II^lacz/lacz^ mice had a normal life span with less severe osteopenia than that in *Runx2*-II^−/−^ mice, and *Runx2*-I was not upregulated (Liu et al., 2011). The expression of *Runx2*-II mRNA was more than three times higher than that of *Runx2*-I in the newborn calvaria (Figure 3G). However, the total *Runx2* mRNA in the calvaria of *Runx2*-II^lacz/lacz^ mice was approximately half that of wild-type mice at the neonatal stage, irrespective of the lack of both *Runx2*-II mRNA and *Runx2*-I upregulation (Liu et al., 2011). Therefore, it is likely that some transcripts from P1 were aberrantly spliced to the downstream of the authentic splicing signal in exon 2 and translated to a functional Runx2 protein, because the translated protein from the second ATG in exon 2 was functional in the first ATG mutated mice (Liu et al., 2011; Okura et al., 2014).

The reduction in *Runx2*-II levels increased *Runx2*-I levels in *Runx2*-br^mut/mut^ mice. There are four Runx motifs upstream of the translation start site of *Runx2*-I in the mouse genome. The upregulation of *Runx2*-I may have been caused by negative autoregulation of its own promoter by Runx2, because the overexpression of *Runx2* reduced P2 luciferase reporter activity (Zhang et al., 2009). However, overexpression of *Runx2* affected neither *Runx2*-I, *Runx2*-II, or total *Runx2* expression (Figure 13B). Furthermore, *Runx2* knockdown failed to affect 1.9-kb P2 luciferase reporter activity (Figures 13C,D). Thus, it is unlikely that the upregulation of *Runx2*-I in *Runx2*-br^mut/mut^ mice was due to negative feedback caused by the reduction in Runx2 expression.

In *Runx2* ^lacz/lacz^ mice, in which IRES-lacz-poly A was inserted in exon 2 and both *Runx2*-I and *Runx2*-II were completely disrupted (Otto et al., 1997), the 5′ untranslated region (UTR) mRNA of *Runx2*-I was upregulated but that of *Runx2*-II was severely downregulated (Zhang et al., 2009). The increase in the 5′UTR mRNA of *Runx2*-I was probably due to the efficient transcription from P2 by the insertion of IRES-lacz-poly A into exon 2. Similarly, in *Runx2*-II^lacz/lacz^ mice, in which exon 1 was replaced with lacZ, lacZ was actively transcribed from P1, suggesting normal or enhanced P1 activity. Thus, all 4 mouse models, including *Runx2* ^lacz/lacz^, *Runx2*-II^−/−^, *Runx2*-II^lacz/lacz^, and *Runx2*-br^mut/mut^ mice, suggest that P1 and P2 activities are reciprocally regulated. As *Runx2* transcription is regulated by multiple enhancers (Matsuo et al., 2025; Kawane et al., 2014), P1 and P2 may compete for binding to enhancers.

In conclusion, Runx2 isoforms had overlapping but preferential properties in bone development. Although the reduction of Runx2-II resulted in an increase in *Runx2*-I in *Runx2*-^brmut/mut^ mice, it was not due to negative feedback caused by Runx2 reduction, but P1 and P2 were likely reciprocally regulated. As the tissue-specific expression of Runx2 is regulated by enhancers (Matsuo et al., 2025), the interaction of P1 and P2 with enhancers requires further investigation. Elucidating the mechanism of transcriptional regulation of *Runx2* is necessary to develop therapies for osteoporosis and fracture healing.

## Data Availability

The original contributions presented in the study are included in the article/Supplementary Material, further inquiries can be directed to the corresponding authors.
