# Supplementary figures and images for: Branch site mutated mice revealed distinct roles of two Runx2 isoforms in bone development
Source: Front Cell Dev Biol. 2026 Apr 24;14:1770176. doi: 10.3389/fcell.2026.1770176 (PMC13152850; doi:10.3389/fcell.2026.1770176)

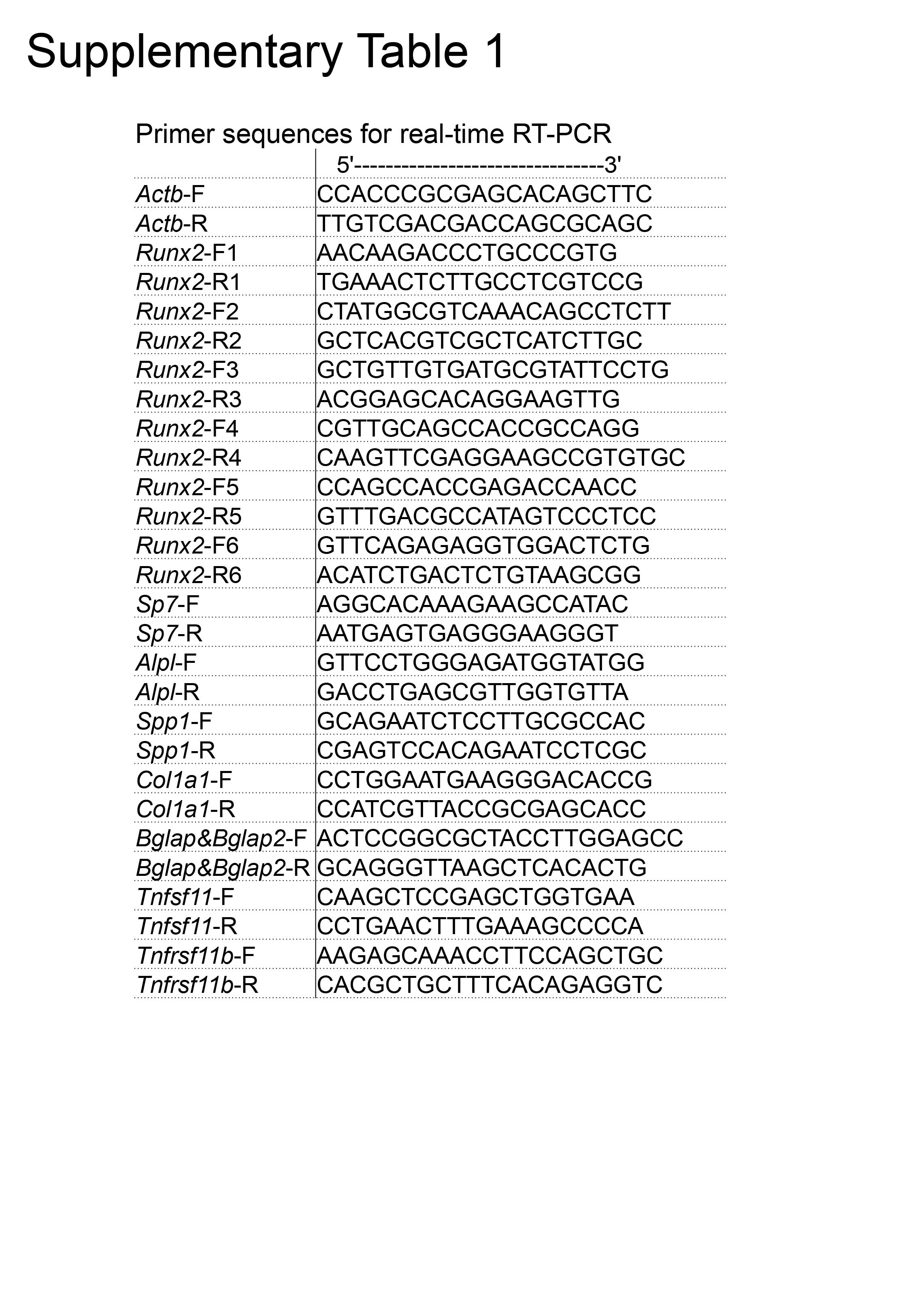

Supplement: Supplementary file 1 [file Image1.jpeg]
